# Nurses’ promotion of Mental Health First Aid Training Programmes for upper secondary students: a modified Delphi approach

**DOI:** 10.1186/s12912-023-01255-3

**Published:** 2023-03-31

**Authors:** Tiago Filipe Oliveira Costa, Antonio Rafael Moreno Poyato, Francisco Miguel Correia Sampaio, María Teresa Lluch Canut, Carlos Alberto da Cruz Sequeira

**Affiliations:** 1 Department of Public Health, Mental Health and Maternal and Child Health Nursing, Nursing School, Universitat de Barcelona, Barcelona, Spain; 2 Centro Hospitalar de Vila Nova de Gaia/Espinho, Vila Nova de Gaia, Portugal; 3 Portuguese Red Cross Northern Health School, Oliveira de Azeméis, Portugal; 4 Center for Health Technology and Services Research of the Health Research Network: From the Lab to the Community (CINTESIS@RISE), Porto, Portugal; 5 Nursing School of Porto, Porto, Portugal; 6 Research Group GEIMAC (Consolidated Group 2014-1139: Group of Studies of Invarianza of the Instruments of Measurement and Analysis of Change in the Social and Health Areas), Barcelona, Spain

**Keywords:** Adolescent, Delphi technique, Education, First Aid, Mental Health, Nursing, Schools

## Abstract

**Background:**

Mental Health First Aid Training Programmes can be carried out by nurses in schools. Adolescents have reported the importance of these interventions, the contents to be addressed, and intervention strategies that should be used. Mental health nurses have also discussed the characteristics of these training programmes. This study sought to create a consensus on the features of Mental Health First Aid Training Programmes promoted by nurses aimed at upper secondary students.

**Methods:**

A descriptive mixed method study was developed using the modified e-Delphi technique. Original statements on the topic were drawn from the results section of previous studies with nurses and adolescents. The statements were included in a structured online questionnaire. An expert panel of 78 mental health nurses participated in the two-rounds online survey from October to December 2021. Absolute and relative frequencies of responses were analysed. The experts’ comments were also considered.

**Results:**

In the first round, experts suggested 4 new ideas about training programmes. Experts took a position on a total of 59 declarations. At least 75% of participants agreed with 58 statements about training programmes, namely about facilitators, intervention foci, evaluation methodology, participants, the implementation context, period and regularity, intervention strategies and content. The experts’ opinions differed only in one statement that expressed the impaired sexual behaviour as a mental health problem to be addressed in the training programmes.

**Conclusions:**

Mental health nurses can lead the delivery of training programmes in upper secondary schools, improving adolescent competencies in mental health. Several educational approaches can be used to teach about mental health, related problems and actions underlying these conditions. Valid and appropriate assessment methods must be used. Our findings guide the planning, implementation and evaluation of these interventions in upper secondary schools and encourage nurses to explore these programmes and include them in their educational curricula.

**Supplementary Information:**

The online version contains supplementary material available at 10.1186/s12912-023-01255-3.

## Background

The prevalence of mental health problems has increased over time. In 1990, approximately 655 million people worldwide were living with a mental disorder and, in 2019, the prevalence was already 970 million people [1]. In this way, the general public is increasingly closer to individuals with mental health problems and has the opportunity to provide first aid. This initial help is usually given by someone from the social network of the person with mental health problems (such as friends and family) and not by mental health professionals. Initial support given to a person with a mental health problem (until professional help arrives or the situation is resolved) can be called Mental Health First Aid [2]. Supporting the use of relaxation strategies and assisting in seeking a mental health nurse are examples of mental health first aid behaviours. According to Wong et al., the suggestion of members of the social network makes individuals with mental health problems more likely to seek professional help [3].

Citizens need to be actively involved in the health of others when providing first aid. The World Health Organization expresses that improving health literacy can promote citizens’ involvement in individual and community health actions [4]. The concept of health literacy comprises the cognitive and social skills needed to access, understand and use the information to promote and maintain good health [5]. Mental health literacy involves knowing strategies to promote positive mental health and how to prevent mental disorders, recognizing mental disorders and the associated stigma, efficacy in seeking help resources and knowing how to provide first aid and support to others [6]. Thus, promoting mental health first aid skills is one way of intervening in fostering mental health literacy [7]. According to Tay et al., low levels of mental health literacy have been reported worldwide [8]. Therefore, Mental Health First Aid Training Programmes are very relevant. These specific interventions aim to empower individuals with basic skills to provide first aid to people with mental health problems [2]. These training programmes value their participants as an informal resource for help.

Several studies have described various interventions with the concept of “Mental Health First Aid Training Programmes”. Costa et al. identified these interventions and their features, participants and implementation contexts. These authors expressed that Mental Health First Aid Training Programmes can target upper secondary students [9]. For example, Hart et al. reported positive outcomes from the classroom implementation of these educational interventions for adolescents aged 15 to 18 years [10].

School is a privileged environment to promote health literacy because it accommodates people from diverse social backgrounds. School interventions are associated with greater cost-effectiveness and positive health outcomes for populations [11]. Regarding Portugal, upper secondary education (level 3 of the International Standard Classification of Education) is universal, compulsory and free. During this educational phase, students must acquire comprehensive knowledge and skills to respond, adapt to situations, and assume responsibilities [12]. Therefore, these school goals overlap with those of Mental Health First Aid Training Programmes.

Portuguese secondary students are generally 15 to 18 years old [12]. Adolescents are expected to achieve hypothetical-deductive, logical and systematic thinking [13]. This achievement allows them to assimilate and accommodate learning. In addition, they are expected to acquire a sense of identity from reflecting on their current and future role in the world [14]. Therefore, this can be a sensitive time for them to incorporate the role of “the first aider”.

The existing literature shows that most Mental Health First Aid Training Programmes address disorders [9]. Such interventions may induce participants to view mental health problems as disorders, promoting the medicalisation and psychiatrisation movement of human suffering [15]. DeFehr indicates that a mental disorder diagnosis is considered by many to be useful and validating, but it can also cause serious harm, such as stigma or stereotype [16]. Therefore, using medical labels to refer to mental health problems in the context of Mental Health First Aid Training Programmes seems inadequate. Alternatively, Costa et al. suggest that mental health nurses develop Mental Health First Aid Training Programmes centred on mental health problems that represent nursing diagnoses (such as anxiety, hallucinations, and substance abuse) [9]. Nursing diagnoses describe human responses to actual or potential health problems and life processes [17]. A nursing diagnosis is a label assigned by a nurse to a decision about a phenomenon that is an area of nursing intervention [18]. These labels are expressed in taxonomies such as the NANDA International’s Nursing Diagnoses and International Classification for Nursing Practice (ICNP) [17, 18]. In contrast, medical diagnoses represent diseases or medical conditions with specific symptoms and signs. These are expressed by terms defined in taxonomies such as the Diagnostic and Statistical Manual of Mental Disorders (DSM) and the International Classification of Diseases (ICD) [19, 20].

In Portugal, school health interventions are carried out by school health teams. These teams are action-oriented in different areas, such as mental health and first aid [21]. The Ordem dos Enfermeiros (the association that regulates the nursing profession in Portugal) recommended the existence of mental health nurses in the context of primary health care [22]. Thus, school health teams are often made up of mental health nurses. Mental health nurses can lead these healthier interventions (not focused on disorders) for adolescents in upper secondary schools. In turn, adolescents can play the role of “first aiders” (people who help others with problems). However, these healthier interventions for upper secondary school students were not found in the literature. Thus, Costa et al. explored the perspective of the target population (adolescent students) and experts (mental health nurses experienced in mental health literacy, working with adolescents aged 15 to 18 years, and within a school environment) on the features of Mental Health First Aid Training Programmes [23, 24]. The opinions of potential facilitators and targets of the intervention were different. For example, adolescents identified some mental health problems that could be addressed in the training programme, such as ambivalence, obsession, impaired sexual behaviour and social isolation [23]. Mental health nurses highlighted other specific problems such as frustration, self-care deficit and impaired communicating act [24]. Thus, it is important to have an expert judgment that considers the ideas of adolescents. In addition, the studies have small samples (12 adolescents and 7 mental health nurses). These participants interacted with each other, influencing their personal opinions. Obtaining judgments from a wider, heterogeneous and quasi-anonymised sample of experts may allow greater validity and reliability in opinions about these training programmes [25]. More valid and reliable views can increase the consistency of interventions’ planning, implementation and evaluation. Therefore, this study has sought to establish a consensus (content validity) on the characteristics of Mental Health First Aid Training Programmes promoted by mental health nurses and aimed at adolescents in Portuguese upper secondary schools. Training programmes’ characteristics include aspects of facilitators, foci of intervention, methodology for assessing the produced outcomes and process, participants, the specific context of implementation, duration and frequency, intervention strategies, and content. Guided by the structure of the UK Medical Research Council for the development and evaluation of complex interventions [26], this work contributes to the modelling of this type of intervention.

## Methods

### Design

The research question was: “What features of these interventions are consensual among experts?”. A descriptive mixed method study was developed using the modified e-Delphi technique [25]. On the one hand, the technique was considered “modified” because the ideas on the topic were obtained from qualitative studies previously carried out with the target population and experts [23, 24]. On the other hand, the technique was deemed “e-Delphi” because the questionnaires run online. This option was taken as it is more convenient for researchers and participants, more economical and easier to manage the data. We consider that the existence of a quantitative component in the study guarantees an objective judgment regarding the characteristics of the intervention. The qualitative component of the study allows for a deeper and more consistent view of the topic. The study was reported following the Recommendations for the Conducting and Reporting of Delphi Studies (CREDES) [27].

### Participants

A non-probabilistic purposive sampling permitted the selection of participants. Experts who fit the following criteria were chosen: to be a Portuguese mental health nurse; to have work experience in mental health literacy (identification of educational needs in mental health, planning, implementation or evaluation of mental health training programmes) with adolescents aged 15 to 18 (direct provision of any nursing care to this population) and/or in a school context (intervention in public or private schools and underlying community); to express willingness and availability to participate in the study.

Costa et al. have described mental health nurses as the most suitable to implement these interventions [8, 24]. Moreover, expertise in this area is likely to produce more pondered and appropriate opinions about the topic. Potential experts were identified by searching the topic in search engines, databases, scientific repositories, books and conference programmes and by analysing the researchers’ contact lists. In addition, the researchers subjectively analysed the expertise of potential participants, considering the quality of work performed and productivity.

An e-mail was sent to the potential participants inviting them to participate in the study. They were asked to complete an online informed consent and a sociodemographic characterization form. The sample size was designed to balance the ability to generate a definitive conclusion and the difficulty of managing a larger panel [25]. Seventy-eight participants were recruited.

### Data collection

**Fig. 1:**
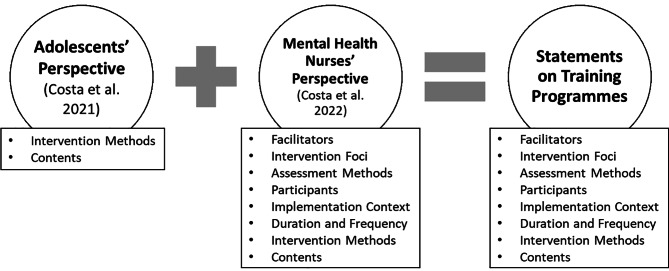
Process followed to create the statements on training programmes

An online structured questionnaire was developed, taking into account the objective of the study (see Additional file 1). The ideas generated in the studies by Costa et al. [23, 24] were listed and compared. Based on these ideas, the researchers created several statements on the topic (see Fig. 1).

The statements were associated with a 5-point Likert agreement scale (1 - Strongly disagree; 2 - Disagree; 3 - Neither agree nor disagree; 4 - Agree; 5 - Strongly agree). The questionnaire also included an open response space, enabling the suggestion of new ideas and the justification of the answers. The questionnaire was pre-tested. The following pre-test questions were asked: “Is the language used in the questions understandable?”, “Is the meaning of the questions clear and unambiguous?”, “Does each question focus on a single topic?”, “Do the questions reflect any bias or “induce” the answers?”, “Is the form of the questions adequate?”. Ten nurses meeting the inclusion criteria completed the questionnaire and reported that it was intelligible and without bias, and no changes were necessary. These ten people were further considered to participate in the following stages of the study.

The questionnaire (see Additional file 1) was sent to the expert panel to fill out. After the first round of responses, a statistical summary of the group’s options (relative frequency of responses) was made available to the experts to consider their individual responses [25]. Then, the questionnaire was revised (see Additional file 2). A second-round questionnaire was produced that included only non-consensual statements and new ideas suggested by the experts in the first round. The frequencies of responses from the first round were appended (when applicable) to each response option in the new questionnaire to facilitate their consideration. After sending and completing the revised questionnaire, a new statistical summary was made available to the participants.

The data collection took place in two response rounds from 12 October to 14 December 2021. The number of response rounds was not defined a priori. One closed round of responses would have been enough if there had been early consensus on all statements and no new ideas had been suggested. Two closed rounds of responses are common practice in the literature because it allows the participants to review their individual opinions while maintaining a high response rate [25]. More than two closed response rounds could have been carried out, but response rates could have decreased. A minimum of a 70% response rate for each round was established to maintain the study’s rigour [25].

### Data analysis

A consensus level of at least 75% was determined a priori [25, 28]. The consensus among experts was considered positive when at least 75% of the responses were classified as 4 or 5 on the Likert scale. There was a negative consensus among experts when at least 75% of the responses were classified as 1 or 2 on the Likert scale. Therefore, the absolute and relative frequencies of the responses were analysed using IBM SPSS Statistics 27 software.

Any statement for which consensus was reached was withdrawn from the next round. This decision was taken because the initial number of statements in the questionnaire was very large. In this way, the questionnaires became shorter as the rounds went on, and the risk of losing expert panel members was reduced [25]. Whenever an open response emerged from the participants, the researchers discussed whether the idea was new or redundant. When a new idea was identified, the researchers wrote a new statement and included it in the questionnaire for the next round.

Whenever consensus was not reached on a sentence, it was submitted to another round. The justifications for the expert opinion were considered whenever consensus was not reached in two consecutive rounds. A content analysis of the justifications of the experts’ options was performed [29, 30]. Units of meaning (set of words with the same central meaning) were identified. They were subjected to a condensation process (text shortening with core preservation). Each unit of meaning was assigned a label (code). In turn, the codes with similarities were grouped and classified into categories (groups of internally homogeneous and externally heterogeneous content). Finally, the categories’ meanings and relationships were interpreted, and themes were formed [29]. No software was used for content analysis. The first author conducted this analysis with the supervision of the others.

### Ethical considerations

The Declaration of Helsinki and the Oviedo Convention for research with human beings were followed throughout the study [31, 32]. The ethics committee of the University of Barcelona (Institutional Review Board: IRB00003099) approved the study.

The participants completed informed consent and took part in the study voluntarily and without compensation. They were informed of the right to withdraw without penalty and access, rectify, limit processing, and delete their data.

The data from each expert was coded with the letter E followed by a number. This coding prevented the attribution of responses to the expert panel members. This way, quasi-anonymity was fulfilled [25], and social pressure and the conformity of responses with a dominant view were avoided.

### Validity, reliability and rigour

The iterative process of the Delphi technique promotes the validity of the results. Setting a response rate of at least 70% supports the study’s validity. The transparency in the methodological choices and the questionnaires’ presentation (see Additional files 1 and 2) ensured the study’s credibility.

The generation of ideas about the topic was separated from this study. However, participants were able to give open responses in the rounds, increasing the reliability of the results. The pre-test of the questionnaires ensured their adequacy. Quasi-anonymity also decreased the risk of bias in participants’ responses. Regarding statements without consensus in the first round of responses, it was mandatory to justify any option taken in the second round. In this way, an attempt was made to reduce the risk of participants having a certain position on the topic simply because other elements of the group have it (bandwagon effect).

The justifications for the lack of consensus in a statement were obtained from a large and heterogeneous sample. In this way, we obtained good coverage of variations in opinion, guaranteeing the credibility of the results. The diversity of characteristics of the study participants and their detailed descriptions also fostered the transferability of the findings. The participation of different authors in the content analysis ensured the rigour of its realization. The presentation of some quotes from the original text, different codes, categories and themes ensured the credibility and authenticity of the results.

## Results

Seventy-eight experts participated in the first round of response (response rate: 100%) and sixty-one experts in the second round (response rate: 78%). Most of the mental health nurses recruited were female (76%). They were aged 25 to 63 years (mean: 43.91; SD: 8.82). Of these, 82% were married or in a non-marital partnership, 10% were single, and 8% were divorced or separated. Most experts had children (79%), but only 17% had children aged 15 to 18. They had different academic backgrounds (21% Licentiate, 53% Master and 27% Doctorate). Some participants were engaged in more than one nursing activity (40%). These activities included care provision (72%), management (12%), education (44%), and research (28%). The nurses were from different regions of mainland Portugal (North (40%), Centre (35%), South (24%), and the Islands (1%)). Their experience as non-specialised nurses ranged from 2 to 31 years (mean: 10.97; SD: 6.32) and as mental health nurses from 0 to 32 years (mean: 10.23; SD: 7.88). It is important to highlight that being a mental health nurse in Portugal requires training in non-specialized nursing plus a specialization course in mental health and psychiatric nursing. The experts also had work experience in the field of mental health literacy (94%), with adolescents aged 15 to 18 years (69%) and in the school context (68%).

**Fig. 2:**
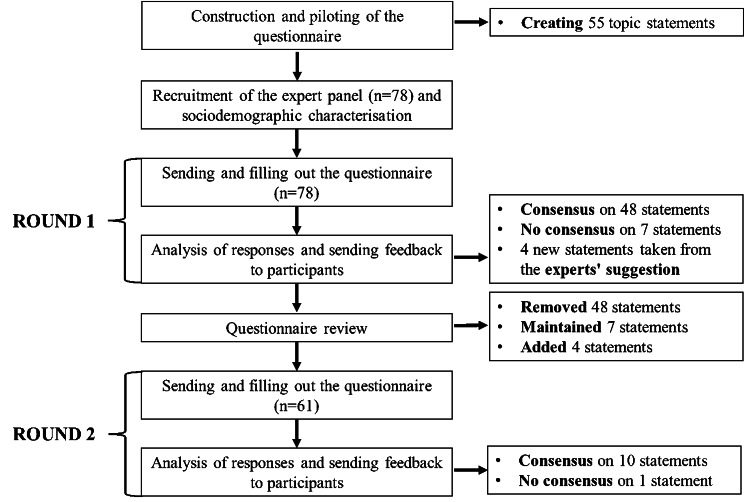
Flowchart of the data collection and analysis

The process of data collection and analysis is summarised in Fig. 2.

Changes in individual experts’ responses from the first to the second round may have occurred by reviewing the responses in light of the group’s opinion. The “neutrality” option was consistently abandoned in the repeated statements in the second round.

### Facilitators

In the first round of responses, the experts agreed that Mental Health First Aid Training Programmes should preferably be promoted by mental health nurses and co-facilitated by professionals with different backgrounds and work contexts (see Table 1).

**Table 1:** Distribution of responses given by the experts about the facilitators of training programmes

Statements	Responses	n (%)
		Round 1
This type of programme should be promoted, preferably, by mental health nurses.	Agreement	73 (94%)*
	Neutral	3 (4%)
	Disagreement	2 (3%)
This type of programme should preferably be carried out with the co-facilitation of professionals with different training (e.g., education, psychology, psychiatry, child and pediatric health, community health) and work contexts (e.g., school, healthcare hospitals, primary care).	Agreement	70 (90%)*
	Neutral	3 (4%)
	Disagreement	5 (6%)

Legend: * - Consensus obtained

### Intervention foci and assessment methods

In the first round of responses, the experts agreed that these training programmes should focus on the participants’ mental health competencies, and the outcomes and intervention processes should be evaluated longitudinally (see Table 2).

**Table 2:** Distribution of responses given by the experts on intervention foci and assessment methods of training programmes

Statements	Responses	n (%)
		Round 1
The intervention foci should be nursing foci related to mental health competencies (e.g., knowledge about first aid aimed at people with mental health problems).	Agreement	76 (97%)*
	Neutral	2 (3%)
	Disagreement	0 (0%)
The intervention outcomes should be evaluated over time with measurement instruments based on nursing knowledge (e.g., indicators contained in the Nursing Outcomes Classification).	Agreement	67 (86%)*
	Neutral	10 (13%)
	Disagreement	1 (1%)
At the end of each session, a formal and verbal assessment of the participants’ satisfaction with the training should be carried out.	Agreement	73 (94%)*
	Neutral	4 (5%)
	Disagreement	1 (1%)
Facilitators should consider the participants’ satisfaction with the training by observing their behaviour.	Agreement	74 (95%)*
	Neutral	4 (5%)
	Disagreement	0 (0%)

Legend: * - Consensus obtained

### Participants

In the first round of responses, the expert panels’ opinions about the participants in the interventions diverged (see Table 3). A consensus was reached on all statements in a second round. The division of classes into groups emerged and was agreed upon as a strategy for managing the number of participants.

**Table 3:** Distribution of responses given by the experts about the participants in training programmes

Statements	Responses	n (%)	
		Round 1	Round 2
Facilitators must implement the intervention for classes (up to 30 students), not excluding participants.	Agreement	58 (74%)	49 (80%)*
	Neutral	12 (15%)	5 (8%)
	Disagreement	8 (10%)	7 (11%)
Facilitators must implement the intervention for classes, being able (whenever necessary) to divide them into groups.	Agreement		57 (93%)*
	Neutral		2 (3%)
	Disagreement		2 (3%)
In the classes, facilitators must identify students with nursing diagnoses related to mental health competencies.	Agreement	58 (74%)	54 (89%)*
	Neutral	15 (19%)	4 (7%)
	Disagreement	5 (6%)	3 (5%)
In the classes to intervene, the facilitators must identify availability and willingness to learn.	Agreement	68 (87%)*	
	Neutral	9 (12%)	
	Disagreement	1 (1%)	
Facilitators should pay special attention to participants who have health problems (physical, mental and/or social).	Agreement	72 (92%)*	
	Neutral	6 (8%)	
	Disagreement	0 (0%)	

Legend: * - Consensus obtained

### Implementation context

In the first round of responses, the experts highlighted the preference for face-to-face delivery of training programmes (see Table 4).

**Table 4:** Distribution of responses given by the experts about the context of the implementation of training programmes

Statements	Responses	n (%)
		Round 1
The implementation of this type of intervention should preferably be in-person, in the classroom and during school time.	Agreement	70 (90%)*
	Neutral	8 (10%)
	Disagreement	0 (0%)
The virtual implementation of this type of intervention should be considered alternative or complementary.	Agreement	59 (76%)*
	Neutral	11 (14%)
	Disagreement	8 (10%)

Legend: * - Consensus obtained

### Duration and frequency

The experts converged in their responses regarding the duration and frequency of the training sessions. However, the statement about the interval between sessions obtained consensus in the second round of responses (see Table 5).

**Table 5:** Distribution of responses given by the experts about the duration and frequency of training programmes

Statements	Responses	n (%)	
		Round 1	Round 2
This type of intervention should have the session duration adjusted to the classes’ duration (commonly 45–90 min).	Agreement	73 (94%)*	
	Neutral	3 (4%)	
	Disagreement	2 (3%)	
The frequency of intervention sessions should be adjusted to the school year and school availability.	Agreement	74 (95%)*	
	Neutral	3 (4%)	
	Disagreement	1 (1%)	
The interval between intervention sessions must be up to one week.	Agreement	54 (69%)	55 (90%)*
	Neutral	18 (23%)	3 (5%)
	Disagreement	6 (8%)	3 (5%)
In this type of intervention, reinforcement/consultation sessions should occur (e.g., at the time of the follow-up assessment).	Agreement	73 (94%)*	
	Neutral	5 (6%)	
	Disagreement	0 (0%)	

Legend: * - Consensus obtained

### Intervention methods and strategies

In the first round of responses, there was agreement on using multiple educational strategies and resources and the importance of awareness sessions for the entire school community (see Table 6).

**Table 6:** Distribution of responses given by the experts about intervention methods and strategies of training programmes

Statements	Responses	n (%)
		Round 1
This type of intervention should use a mixture of different training strategies: expository (e.g., lectures), demonstrative, participatory (e.g., discussions), contact-based education (e.g., testimonials) and experimental learning (e.g., role plays).	Agreement	76 (97%)*
	Neutral	2 (3%)
	Disagreement	0 (0%)
This type of intervention should use multiple educational resources, including informational, technological (e.g., internet, mobile) and audiovisual (e.g., music, video) materials.	Agreement	77 (99%)*
	Neutral	1 (1%)
	Disagreement	0 (0%)
Although this type of intervention targets upper secondary school students, awareness sessions for the school community on the subject should be carried out.	Agreement	76 (97%)*
	Neutral	2 (3%)
	Disagreement	0 (0%)

Legend: * - Consensus obtained

### Contents

The experts agreed on the statements on mental health and its influencing factors and self-help strategies in the first round of responses (see Table 7).

**Table 7:** Distribution of responses given by the experts about the content of training programmes (aspects related to mental health and self-help)

Statements	Responses	n (%)
		Round 1
This type of intervention should address the concept of health and mental health, self-care / self-help / coping strategies.	Agreement	77 (99%)*
	Neutral	1 (1%)
	Disagreement	0 (0%)
Participants can be taught the following examples of self-help strategies: establishing routines; changing environment; making a complete, varied and balanced diet; sleeping regularly; planning leisure and distraction activities; practising regular exercise; reading and writing (including about the problem and helps); perform plastic expression activities; perform body expression activities (theatre, dance); sing; listen to music/audio; perform relaxation and meditation strategies; practice religious worship; volunteering; socialise.	Agreement	76 (97%)*
	Neutral	2 (3%)
	Disagreement	0 (0%)
This type of intervention should address mental health influencing factors (risk and protective factors for mental health problems).	Agreement	75 (96%)*
	Neutral	3 (4%)
	Disagreement	0 (0%)

Legend: * - Consensus obtained

Furthermore, the experts agreed on the importance of addressing aspects concerning mental health problems (see Table 8). It was suggested to highlight the stigma affecting people with mental health problems in the first round of responses, and a statement on the subject was agreed upon in the second round.

**Table 8:** Distribution of responses given by the experts about the content of training programmes (aspects related to mental health problems)

Statements	Responses	n (%)	
		Round 1	Round 2
This type of intervention should address mental health problems and mental disorders, namely their concepts, examples, incidence and prevalence rates, main manifestations (signs and symptoms), experiences and consequences related to mental health problems.	Agreement	62 (79%)*	
	Neutral	12 (15%)	
	Disagreement	4 (5%)	
These types of programmes should address stigma as an experience lived by people with mental health problems.	Agreement		54 (89%)*
	Neutral		2 (3%)
	Disagreement		5 (8%)
In this type of training programme, cognitive, behavioural, emotional and relational problems experienced by the general public must be addressed.	Agreement	68 (87%)*	
	Neutral	8 (10%)	
	Disagreement	2 (3%)	

Legend: * - Consensus obtained

The experts also reached a consensus on most statements about mental health problems to be specifically addressed in training programmes (see Additional file 3). As shown in Additional file 3, there was no consensus in two consecutive rounds in the statement “Behavioural problems to be addressed in training programmes include problems in sexual behaviour (impaired sexual behaviour [paraphilias])”. On the one hand, “Promotion of competencies to act on a problem relevant to the participants” was the justification for the agreement between experts regarding the statement (see Additional file 4). For example, E57 explained that paraphilias are an “Important topic in adolescence and in discovering sexuality”. E68 added that “it is a matter of interest to adolescents”.

On the other hand, “Inappropriate learning for adolescents” was the reason for disagreement (see Additional file 4). For example, E28 indicated, “I don’t think this topic is essential in the Mental Health First Aid Training Programme”. E66 added, “I don’t know if addressing sexual problems can somehow influence the adolescent’s experience, who is at the peak of his/her discovery”.

In the first round of responses, the experts reached a consensus on the contents of the training programmes about providing help to people with mental health problems. In addition, they highlighted the importance of exploring communication and interpersonal relationships and the solidarity and civic ethics underlying the aider’s role. Thus, the statements on the matter were created and agreed upon in the second round. Table 9 presents this distribution of responses.

**Table 9:** Distribution of responses given by the experts about the content of training programmes (aspects concerning the provision of help to people with mental health problems)

Statements	Responses	n (%)	
		Round 1	Round 2
These programmes should address the need for help, facilitating factors, and barriers to seeking help.	Agreement	77 (99%)*	
	Neutral	0 (0%)	
	Disagreement	1 (1%)	
These types of programmes should explore communication and interpersonal relationships.	Agreement		60 (98%)*
	Neutral		1 (2%)
	Disagreement		0 (0%)
These training programmes should address mental health first aid, namely its concept, importance, participants, implementation contexts and modes of action.	Agreement	77 (99%)*	
	Neutral	1 (1%)	
	Disagreement	0 (0%)	
These types of programmes should address the solidarity and civic ethics underlying the aider’s role.	Agreement		56 (92%)*
	Neutral		5 (8%)
	Disagreement		0 (0%)
These training programmes should address key informal and formal resources for help.	Agreement	78 (100%)*	
	Neutral	0 (0%)	
	Disagreement	0 (0%)	
These training programmes should highlight help from family and social network members, school staff, local health services (nurses, psychologists, doctors, self-help and mutual aid groups) and distance health services (virtual therapies, telephone helplines).	Agreement	78 (100%)*	
	Neutral	0 (0%)	
	Disagreement	0 (0%)	
These training programmes should teach participants a dynamic action plan with the following steps: approach the person and assess the situation; assist and encourage the person to use self-help strategies; assist and encourage the person to seek formal and informal help; take care of oneself (first aider).	Agreement	77 (99%)*	
	Neutral	1 (1%)	
	Disagreement	0 (0%)	

Legend: * - Consensus obtained

The experts also obtained agreement on all statements regarding mental health first aid actions to be proposed in training programmes (see Additional file 3).

## Discussion

This study sought to reach a consensus on the characteristics of Mental Health First Aid Training Programmes promoted by nurses and aimed at adolescents in upper secondary schools. The experts expressed their position regarding the facilitators of training programmes, intervention foci, outcomes and process assessment strategies, participants, implementation context, duration and frequency, intervention strategies, and contents. Our results show that experts agree on most of the ideas about the intervention, generated by them and in the qualitative studies by Costa et al. [23, 24]. Only impaired sexual behaviour did not obtain consensus as the training programme’s content. In the literature, there does not seem to be consistent evidence about the appropriateness of talking about sexual deviance with adolescents and its potential influence.

The importance of professionals from different areas of training and work contexts participating in the implementation of these training programmes was highlighted. Fleury et al. indicate that multidisciplinary teams working in several health areas can be more beneficial to users when compared with care provided by a single professional [33]. Facilitators with diverse backgrounds can foster a complementary and successful approach.

It was agreed that these programmes respond to nursing foci (relevant care areas for nurses) related to mental health competencies. Thus, the experts also highlighted the need to identify diagnoses of this type in the participants that may justify the implementation of the intervention. According to Salman et al., competence involves knowledge, skill, attitude, performance and task execution and can be improved with training [34]. The availability and willingness of participants to learn were considered critical for implementing training programmes. Frey expresses that the motivation of adolescents influences their performance and involvement in the classroom [35].

The relevance of evaluating interventions over time with valid and appropriate methods was also consensual. The evaluation of outcomes determines the effectiveness and usefulness of the intervention [26]. Instruments that assess mental health competencies (intervention focus) can be considered. Educational needs and the gains obtained from the intervention can be determined by the participants and/or facilitators. Scales that measure the participants’ real competencies and those perceived by them may be relevant. However, the evaluated construct should have a healthier perspective similar to the training programme. Potential limitations in the intervention can also be hypothesized due to its ineffectiveness. The evaluation of the process allows for identifying and improving less positive aspects of the intervention [26]. The satisfaction verbalized by the participants and observed in their behaviour, and the aspects recognized by them as more and less positive in the intervention can be relevant to detect gaps and adjust the training programme. A checklist and a questionnaire with open and closed questions can be prepared for this purpose. The adoption of assessment instruments should be based on evidence of their psychometric properties and the feasibility of their use [36].

The experts agreed that interventions should be carried out according to the existing groups. In schools, students are commonly organized into groups called classes. Classes are more likely to be consolidated groups and already in a phase of “work and productivity” [37]. Moreover, it is easier to access the participants in their usual organization. Thus, the implementation of the intervention can be more efficient. School time was also considered adequate for implementing training programmes. School times are predetermined periods for learning. Therefore, school availability must be considered, and flexible intervention plans must be provided to facilitate the operationalisation of training programmes.

The experts preferred face-to-face education to virtual learning. Both modes of delivery of training have advantages and disadvantages. Gherhes et al. explain that face-to-face education seems to have more impact than virtual learning [38]. Face-to-face learning facilitates interaction between trainers and trainees and between trainees. However, it is important to emphasise the flexibility and low cost of virtual education.

The experts agreed that varied training strategies and educational resources should be used in the interventions. Mixing teaching methods can be useful, as all strategies have advantages and disadvantages [39]. Although the target of the intervention was adolescents, the experts confirmed the importance of raising awareness in the school community about mental health issues. According to Spier et al., families and communities have an important influence on the education of adolescents [40].

The experts agreed on the importance of addressing mental health, influencing factors and self-help strategies in training programmes. The concept of mental health must be seen as a continuum: being well, having a mental health problem, having a mental disorder and being recovered [2]. In this continuum, the mental health of individuals can always be promoted [41]. In this way, these learning experiences can be useful for adolescents, enabling them to self-manage their mental health status, even after helping another person.

The experts agreed that training programmes should mainly address mental health problems and aspects of helping people experiencing them. The consensual mental health problems were emotional, cognitive, behavioural and relational, in line with those addressed in existing training programmes [9]. However, they reflected nursing diagnoses listed in nursing taxonomies [17, 18]. The problems to be deepened in each context must consider the educational needs of the participants. Concerning helping people with mental health problems, it was agreed that participants need to learn about seeking help resources and providing mental health first aid. Gulliver et al. express the factors influencing help-seeking: the level of mental health literacy, recognition of the mental health problem, beliefs regarding mental disorders and seeking help, accessibility, previous experiences and relationship to help resources [42]. Kitchener et al. describe that Mental Health First Aid Training Programmes teach how to recognise signs and value symptoms of mental health problems to provide adequate initial help and refer them to professionals and other support [2]. Therefore, the first aid actions and information supporting them must be explored.

This study had some limitations that should be highlighted. Although the attrition rate between rounds was low, it may have influenced the results. The removal of the statements in the questionnaires after consensus also made it impossible to observe the stability of the responses in different rounds. An intrinsic limitation of the Delphi technique is the risk of the bandwagon effect.

The consensus on the facilitation of training programmes by mental health nurses should be viewed with caution, as the panel of experts was only made up of nursing professionals. In addition, the sample intentionally had particular characteristics. Therefore, it cannot be considered representative of all mental health nurses in Portugal.

## Conclusion

Experts reached a consensus on the characteristics of Mental Health First Aid Training Programmes for upper secondary students. They agreed that mental health nurses can promote these training programmes, improving adolescents’ mental health competencies. A valid evaluation of the process and the outcomes of the interventions is essential over time. They considered that different educational methods should be used in the classroom to teach adolescents to act towards people with mental health problems. Several cognitive, behavioural, emotional and relational problems should be addressed. Research on teaching adolescents about impaired sexual behaviours/paraphilias should be carried out to support the adoption or not of this content in training programmes.

This report provides a “consensual outline” of this type of training programme promoted by nurses. However, feasibility and piloting studies of the interventions must be carried out. The need for some adjustment in the characteristics of the interventions can be identified by testing the procedures, estimating the recruitment and retention of participants and determining the sample size. Furthermore, it is important to identify appropriate assessment instruments to represent the process and results of interventions. Literature reviews on assessment tools should be considered. Studies to develop/adapt assessment instruments and explore their psychometric properties should be considered.

The evidence obtained may instigate mental health nurses to lead Mental Health First Aid Training Programmes in schools. In addition, it can encourage the collaboration of mental health nurses with professionals from other disciplines (e.g., psychologists, physicians/psychiatrists, teachers) and with other nurses (non-specialists or non-specialists in mental health) in the promotion of these interventions. The nursing education curriculum should raise awareness about these Mental Health First Aid Training Programmes, as any nurse can participate in their implementation or assist people with mental health problems. The education curriculum for mental health nurses must ensure that these professionals consistently implement these training programmes.

This study systematises the characteristics of the training programmes. Therefore, it is an asset for professionals working in schools, as it guides the development of this type of intervention. It highlights the mental health problems representing nursing diagnoses that should be addressed in these educational actions. Adolescents who these training programmes will target will be able to recognise, help and refer people with mental health problems without inappropriately assigning “medical labels”.

## Electronic supplementary material

Below is the link to the electronic supplementary material.

Supplementary Material 1

Supplementary Material 2

Supplementary Material 3

Supplementary Material 4

## Data Availability

The datasets generated and/or analysed during the current study are not publicly available due to privacy or ethical restrictions but are available from the corresponding author on reasonable request.
