# Supplementary material for: Nurses’ promotion of Mental Health First Aid Training Programmes for upper secondary students: a modified Delphi approach
Source: BMC Nurs. 2023 Mar 31;22:91. doi: 10.1186/s12912-023-01255-3 (PMC10064774; doi:10.1186/s12912-023-01255-3)
Supplement: Supplementary file 1 — Supplementary Material 1 [file 12912_2023_1255_MOESM1_ESM.docx]

**Additional file 1** (.docx) - First round questionnaire content.

Questionnaire content used in the first Delphi round is presented. It was translated from European Portuguese to English.

**Mental Health First Aid Training Programmes** are interventions that aim to **empower their participants** to provide first aid to people with mental health problems. Nurses can carry out these interventions for adolescents in upper secondary schools. In turn, adolescents can play the role of "first aiders" (people who help others with problems).

**
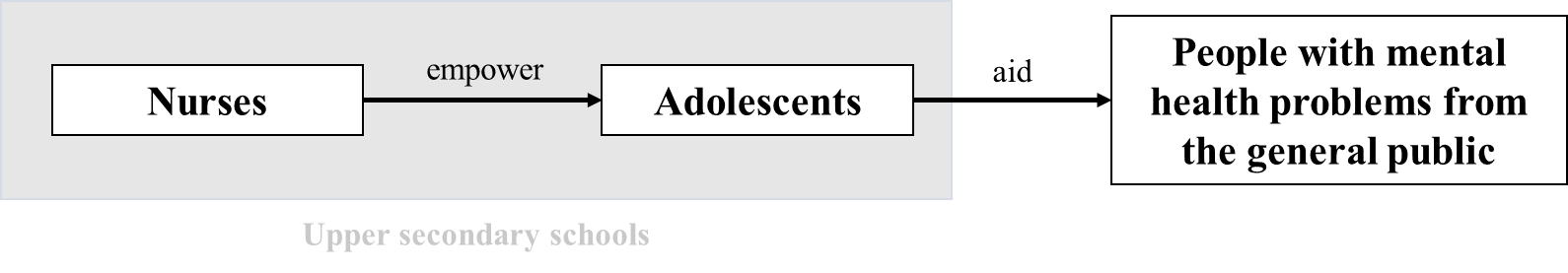
**

In this section of the form, statements regarding Mental Health First Aid Training Programmes promoted by nurses and aimed at adolescents in Portuguese upper secondary schools are presented. Please select your level of agreement (1 - Strongly disagree, 2 – Disagree, 3 - Neither agree nor disagree, 4 – Agree, 5 - Strongly agree) from the statements below.

**Facilitators**

- This type of programme should be promoted, preferably, by mental health nurses.

| 1 | 2 | 3 | 4 | 5 |
| --- | --- | --- | --- | --- |

- This type of programme should preferably be carried out with the co-facilitation of professionals with different training (e.g., education, psychology, psychiatry, child and pediatric health, community health) and work contexts (e.g., school, healthcare hospitals, primary care).

| 1 | 2 | 3 | 4 | 5 |
| --- | --- | --- | --- | --- |

**Intervention Foci**

- The intervention foci should be nursing foci related to mental health competencies (e.g., knowledge about first aid aimed at people with mental health problems).

| 1 | 2 | 3 | 4 | 5 |
| --- | --- | --- | --- | --- |

**Outcomes Assessment Methods**

- The intervention outcomes should be evaluated over time with measurement instruments based on nursing knowledge (e.g., indicators contained in the Nursing Outcomes Classification).

| 1 | 2 | 3 | 4 | 5 |
| --- | --- | --- | --- | --- |

**Process Assessment Methods**

- At the end of each session, a formal and verbal assessment of the participants’ satisfaction with the training should be carried out.

| 1 | 2 | 3 | 4 | 5 |
| --- | --- | --- | --- | --- |

- Facilitators should consider the participants’ satisfaction with the training by observing their behaviour.

| 1 | 2 | 3 | 4 | 5 |
| --- | --- | --- | --- | --- |

**Participants**

- Facilitators must implement the intervention for classes (up to 30 students), not excluding participants.

| 1 | 2 | 3 | 4 | 5 |
| --- | --- | --- | --- | --- |

- In the classes, facilitators must identify students with nursing diagnoses related to mental health competencies.

| 1 | 2 | 3 | 4 | 5 |
| --- | --- | --- | --- | --- |

- In the classes to intervene, the facilitators must identify availability and willingness to learn.

| 1 | 2 | 3 | 4 | 5 |
| --- | --- | --- | --- | --- |

- Facilitators should pay special attention to participants who have health problems (physical, mental and/or social).

| 1 | 2 | 3 | 4 | 5 |
| --- | --- | --- | --- | --- |

**Implementation Context**

- The implementation of this type of intervention should preferably be in-person, in the classroom and during school time.

| 1 | 2 | 3 | 4 | 5 |
| --- | --- | --- | --- | --- |

- The virtual implementation of this type of intervention should be considered alternative or complementary.

| 1 | 2 | 3 | 4 | 5 |
| --- | --- | --- | --- | --- |

**Duration and Frequency**

- This type of intervention should have the session duration adjusted to the classes' duration (commonly 45-90 minutes).

| 1 | 2 | 3 | 4 | 5 |
| --- | --- | --- | --- | --- |

- The frequency of intervention sessions should be adjusted to the school year and school availability.

| 1 | 2 | 3 | 4 | 5 |
| --- | --- | --- | --- | --- |

- The interval between intervention sessions must be up to one week.

| 1 | 2 | 3 | 4 | 5 |
| --- | --- | --- | --- | --- |

- In this type of intervention, reinforcement/consultation sessions should occur (e.g., at the time of the follow-up assessment).

| 1 | 2 | 3 | 4 | 5 |
| --- | --- | --- | --- | --- |

**Intervention Methods and Strategies**

- This type of intervention should use a mixture of different training strategies: expository (e.g., lectures), demonstrative, participatory (e.g., discussions), contact-based education (e.g., testimonials) and experimental learning (e.g., role plays).

| 1 | 2 | 3 | 4 | 5 |
| --- | --- | --- | --- | --- |

- This type of intervention should use multiple educational resources, including informational, technological (e.g., internet, mobile) and audiovisual (e.g., music, video) materials.

| 1 | 2 | 3 | 4 | 5 |
| --- | --- | --- | --- | --- |

- Although this type of intervention targets upper secondary school students, awareness sessions for the school community on the subject should be carried out.

| 1 | 2 | 3 | 4 | 5 |
| --- | --- | --- | --- | --- |

**Contents**

- This type of intervention should address the concept of health and mental health, self-care / self-help / coping strategies.

| 1 | 2 | 3 | 4 | 5 |
| --- | --- | --- | --- | --- |

- Participants can be taught the following examples of self-help strategies: establishing routines; changing environment; making a complete, varied and balanced diet; sleeping regularly; planning leisure and distraction activities; practising regular exercise; reading and writing (including about the problem and helps); perform plastic expression activities; perform body expression activities (theatre, dance); sing; listen to music/audio; perform relaxation and meditation strategies; practice religious worship; volunteering; socialise.

| 1 | 2 | 3 | 4 | 5 |
| --- | --- | --- | --- | --- |

- This type of intervention should address mental health influencing factors (risk and protective factors for mental health problems).

| 1 | 2 | 3 | 4 | 5 |
| --- | --- | --- | --- | --- |

- This type of intervention should address mental health problems and mental disorders, namely their concepts, examples, incidence and prevalence rates, main manifestations (signs and symptoms), experiences and consequences related to mental health problems.

| 1 | 2 | 3 | 4 | 5 |
| --- | --- | --- | --- | --- |

- In this type of training programme, cognitive, behavioural, emotional and relational problems experienced by the general public must be addressed.

| 1 | 2 | 3 | 4 | 5 |
| --- | --- | --- | --- | --- |

- Cognitive problems to be addressed in training programmes include memory problems (impaired memory).

| 1 | 2 | 3 | 4 | 5 |
| --- | --- | --- | --- | --- |

- Cognitive problems to be addressed in training programmes include attention problems (impaired attention, impaired concentration).

| 1 | 2 | 3 | 4 | 5 |
| --- | --- | --- | --- | --- |

- Cognitive problems to be addressed in training programmes include language problems (dyslexia, dysphasia).

| 1 | 2 | 3 | 4 | 5 |
| --- | --- | --- | --- | --- |

- Cognitive problems to be addressed in training programmes include problems of perception (hallucination).

| 1 | 2 | 3 | 4 | 5 |
| --- | --- | --- | --- | --- |

- Cognitive problems to be addressed in training programmes include thinking problems (impaired thinking, impaired learning, suicidal ideation, obsession, delirium, dementia, confusion).

| 1 | 2 | 3 | 4 | 5 |
| --- | --- | --- | --- | --- |

- Cognitive problems to be addressed in training programmes include impaired beliefs (negative self-image, disturbed personal identity, disturbed body image, low self-esteem, excessive self-esteem).

| 1 | 2 | 3 | 4 | 5 |
| --- | --- | --- | --- | --- |

- Behavioural problems to be addressed in training programmes include self-care problems (self-care deficit).

| 1 | 2 | 3 | 4 | 5 |
| --- | --- | --- | --- | --- |

- Behavioural problems to be addressed in training programmes include problems with compulsions (compulsive behaviour).

| 1 | 2 | 3 | 4 | 5 |
| --- | --- | --- | --- | --- |

- Behavioural problems to be addressed in training programmes include eating problems (compulsive eating behaviour, impaired eating behaviour [anorexia], bulimia).

| 1 | 2 | 3 | 4 | 5 |
| --- | --- | --- | --- | --- |

- Behavioural problems to be addressed in training programmes include problems in the pattern of exercise (impaired exercise behaviour, excessive physical exercise).

| 1 | 2 | 3 | 4 | 5 |
| --- | --- | --- | --- | --- |

- Behavioural problems to be addressed in training programmes include substance use problems (alcohol abuse, drug abuse, tobacco abuse).

| 1 | 2 | 3 | 4 | 5 |
| --- | --- | --- | --- | --- |

- Behavioural problems to be addressed in training programmes include problems with gambling, video games and internet ([gambling] abuse, [video games] abuse, [internet] abuse).

| 1 | 2 | 3 | 4 | 5 |
| --- | --- | --- | --- | --- |

- Behavioural problems to be addressed in training programmes include aggressiveness problems (aggressive behaviour, aggressive behaviour [harassment], violence, [cybernetic] violence, [sexual] violence).

| 1 | 2 | 3 | 4 | 5 |
| --- | --- | --- | --- | --- |

- Behavioural problems to be addressed in training programmes include self-injurious problems (self-destructive behaviour, self-mutilation, attempted suicide).

| 1 | 2 | 3 | 4 | 5 |
| --- | --- | --- | --- | --- |

- Behavioural problems to be addressed in training programmes include problems in sexual behaviour (impaired sexual behaviour [paraphilias]).

| 1 | 2 | 3 | 4 | 5 |
| --- | --- | --- | --- | --- |

- Behavioural problems to be addressed in training programmes include sleep problems (impaired sleep, insomnia).

| 1 | 2 | 3 | 4 | 5 |
| --- | --- | --- | --- | --- |

- Behavioural problems to be addressed in training programmes include problems in the organisation of behaviours (disorganised behaviour).

| 1 | 2 | 3 | 4 | 5 |
| --- | --- | --- | --- | --- |

- Behavioural problems to be addressed in training programmes include problems with the frequency of behaviours (hyperactivity).

| 1 | 2 | 3 | 4 | 5 |
| --- | --- | --- | --- | --- |

- Emotional problems to be addressed in training programmes include the existence of negative emotions (anxiety, nervousness, sadness, depressed mood, grief, loneliness, ambivalence, euphoria, fear, trauma, stress, fatigue, exhaustion, distress, despair, jealousy, guilt, frustration, insecurity, envy, anger, powerlessness, suffering, shame).

| 1 | 2 | 3 | 4 | 5 |
| --- | --- | --- | --- | --- |

- Emotional problems to be addressed in training programmes include lack of positive emotions (lack of hope, lack of trust, lack of pride, lack of pleasure).

| 1 | 2 | 3 | 4 | 5 |
| --- | --- | --- | --- | --- |

- Relational problems to be addressed in training programmes include problems of socialisation (social isolation, impaired socialisation).

| 1 | 2 | 3 | 4 | 5 |
| --- | --- | --- | --- | --- |

- Relational problems to be addressed in training programmes include communication problems (impaired communicating act).

| 1 | 2 | 3 | 4 | 5 |
| --- | --- | --- | --- | --- |

- These programmes should address the need for help, facilitating factors, and barriers to seeking help.

| 1 | 2 | 3 | 4 | 5 |
| --- | --- | --- | --- | --- |

- These training programmes should address mental health first aid, namely its concept, importance, participants, implementation contexts and modes of action.

| 1 | 2 | 3 | 4 | 5 |
| --- | --- | --- | --- | --- |

- These training programmes should address key informal and formal resources for help.

| 1 | 2 | 3 | 4 | 5 |
| --- | --- | --- | --- | --- |

- These training programmes should highlight help from family and social network members, school staff, local health services (nurses, psychologists, doctors, self-help and mutual aid groups) and distance health services (virtual therapies, telephone helplines).

| 1 | 2 | 3 | 4 | 5 |
| --- | --- | --- | --- | --- |

- These training programmes should teach participants a dynamic action plan with the following steps: approach the person and assess the situation; assist and encourage the person to use self-help strategies; assist and encourage the person to seek formal and informal help; take care of oneself (first aider).

| 1 | 2 | 3 | 4 | 5 |
| --- | --- | --- | --- | --- |

- In the step “Approach the person and assess the situation”, an aider can: introduce yourself; explain the purpose of the aider's presence; ensure confidentiality; express availability (to listen, be present and help); be present; actively listening with an expression of interest, respect, understanding and without judgement; assess the person's cultural background; observe behaviour; coordinate behaviour with the person being helped; physically approach the person, ensuring their safety; accompany the person to a quiet, safe, comfortable place that allows privacy; ask about the situation; ask about the problem, causes, aggravating and mitigating factors and consequences.

| 1 | 2 | 3 | 4 | 5 |
| --- | --- | --- | --- | --- |

- In the step “Assist and encourage the person to use self-help strategies”, an aider can: assist in identifying the mental health problem; inform about the mental health problem; assist in identifying adaptive strategies; encourage the use of adaptive strategies; praise adaptive strategies already in use; encourage abandonment of maladaptive strategies.

| 1 | 2 | 3 | 4 | 5 |
| --- | --- | --- | --- | --- |

- In the step “Assist and encourage the person to seek formal and informal help”, an aider can: assist in the seeking for informal and formal help; inform about the role of help resources; accompany in the seeking for informal and formal help; asking for help in an extreme situation; encourage the seeking for informal and formal help.

| 1 | 2 | 3 | 4 | 5 |
| --- | --- | --- | --- | --- |

- In the step “Take care of oneself (first aider)”, an aider can use adaptive self-help strategies and, if necessary, can seek help for themselves.

| 1 | 2 | 3 | 4 | 5 |
| --- | --- | --- | --- | --- |

If you consider it pertinent, you can make comments, suggest changes to previously presented statements, or even justify your position in the responses given: *(open and optional response)*
