# Supplementary material for: Nurses’ promotion of Mental Health First Aid Training Programmes for upper secondary students: a modified Delphi approach
Source: BMC Nurs. 2023 Mar 31;22:91. doi: 10.1186/s12912-023-01255-3 (PMC10064774; doi:10.1186/s12912-023-01255-3)
Supplement: Supplementary file 2 — Supplementary Material 2 [file 12912_2023_1255_MOESM2_ESM.docx]

**Additional file 2** (.docx) - Second round questionnaire content

Questionnaire content used in the second Delphi round is presented. It was translated from European Portuguese to English.

**Mental Health First Aid Training Programmes** are interventions that aim to **empower their participants** to provide first aid to people with mental health problems. Nurses can carry out these interventions for adolescents in upper secondary schools. In turn, adolescents can play the role of "first aiders" (people who help others with problems).


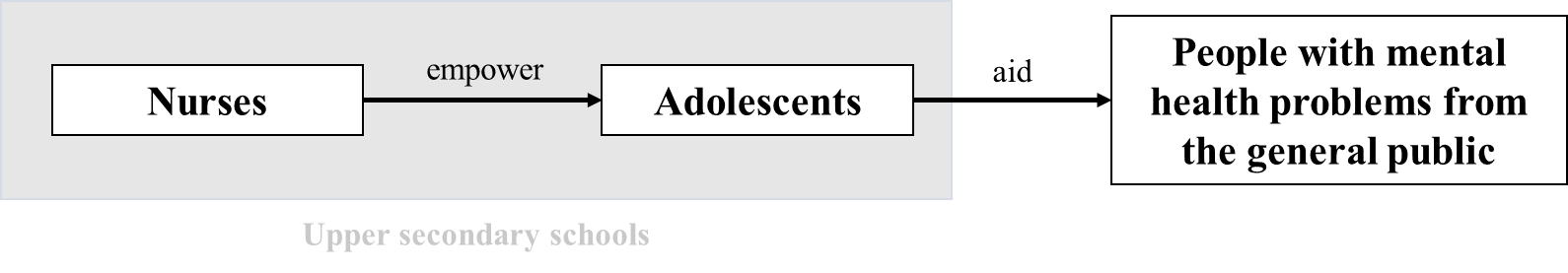


In the form previously sent, statements were presented regarding mental health first aid training programmes promoted by nurses and aimed at adolescents in Portuguese upper secondary schools. Experts were given the opportunity to express their level of agreement with the statements. Consensus was created on several statements. In this new form, the statements where it was not possible to obtain a consensus (positive or negative) were maintained, and other statements resulting from the suggestions given by the participants were added.

Please select your level of agreement from the statements below. The statements will have an **indication of the global distribution of the responses** previously given by the participants.

| Facilitators must implement the intervention for classes (up to 30 students), not excluding participants.  1 - Strongly disagree (prior response from 1% of the group)  2 - Disagree (prior response from 9% of the group)  3 - Neither agree nor disagree (prior response from 15% of the group)  4 - Agree (prior response from 44% of the group)  5 - Strongly agree (prior response from 31% of the group) |
| --- |
| Please justify your choice: *(open and mandatory response)* |

| Facilitators must implement the intervention for classes, being able (whenever necessary) to divide them into groups.  1 - Strongly disagree  2 - Disagree  3 - Neither agree nor disagree  4 - Agree  5 - Strongly agree |
| --- |
| Please justify your choice (optional answer): *(open response)* |

| In the classes, facilitators must identify students with nursing diagnoses related to mental health competencies.  1 - Strongly disagree (prior response from 0% of the group)  2 - Disagree (prior response from 6% of the group)  3 - Neither agree nor disagree (prior response from 19% of the group)  4 - Agree (prior response from 35% of the group)  5 - Strongly agree (prior response from 40% of the group) |
| --- |
| Please justify your choice: *(open and mandatory response)* |

| The interval between intervention sessions must be up to one week.  1 - Strongly disagree (prior response from 3% of the group)  2 - Disagree (prior response from 5% of the group)  3 - Neither agree nor disagree (prior response from 23% of the group)  4 - Agree (prior response from 36% of the group)  5 - Strongly agree (prior response from 33% of the group) |
| --- |
| Please justify your choice: *(open and mandatory response)* |

| These types of programmes should address stigma as an experience lived by people with mental health problems.  1 - Strongly disagree  2 - Disagree  3 - Neither agree nor disagree  4 - Agree  5 - Strongly agree |
| --- |
| Please justify your choice (optional answer): *(open response)* |

| These types of programmes should address the solidarity and civic ethics underlying the aider's role.  1 - Strongly disagree  2 - Disagree  3 - Neither agree nor disagree  4 - Agree  5 - Strongly agree |
| --- |
| Please justify your choice (optional answer): *(open response)* |

| These types of programmes should explore communication and interpersonal relationships.  1 - Strongly disagree  2 - Disagree  3 - Neither agree nor disagree  4 - Agree  5 - Strongly agree |
| --- |
| Please justify your choice (optional answer): *(open response)* |

| Cognitive problems to be addressed in training programmes include memory problems (impaired memory).  1 - Strongly disagree (prior response from 5% of the group)  2 - Disagree (prior response from 8% of the group)  3 - Neither agree nor disagree (prior response from 26% of the group)  4 - Agree (prior response from 32% of the group)  5 - Strongly agree (prior response from 30% of the group) |
| --- |
| Please justify your choice: *(open and mandatory response)* |

| Cognitive problems to be addressed in training programmes include language problems (dyslexia, dysphasia).  1 - Strongly disagree (prior response from 6% of the group)  2 - Disagree (prior response from 6% of the group)  3 - Neither agree nor disagree (prior response from 28% of the group)  4 - Agree (prior response from 32% of the group)  5 - Strongly agree (prior response from 27% of the group) |
| --- |
| Please justify your choice: *(open and mandatory response)* |

| Cognitive problems to be addressed in training programmes include problems of perception (hallucination).  1 - Strongly disagree (prior response from 6% of the group)  2 - Disagree (prior response from 12% of the group)  3 - Neither agree nor disagree (prior response from 15% of the group)  4 - Agree (prior response from 33% of the group)  5 - Strongly agree (prior response from 33% of the group) |
| --- |
| Please justify your choice: *(open and mandatory response)* |

| Behavioural problems to be addressed in training programmes include problems in sexual behaviour (impaired sexual behaviour [paraphilias]).  1 - Strongly disagree (prior response from 6% of the group)  2 - Disagree (prior response from 6% of the group)  3 - Neither agree nor disagree (prior response from 13% of the group)  4 - Agree (prior response from 49% of the group)  5 - Strongly agree (prior response from 26% of the group) |
| --- |
| Please justify your choice: *(open and mandatory response)* |
