# Supplementary material for: Nurses’ promotion of Mental Health First Aid Training Programmes for upper secondary students: a modified Delphi approach
Source: BMC Nurs. 2023 Mar 31;22:91. doi: 10.1186/s12912-023-01255-3 (PMC10064774; doi:10.1186/s12912-023-01255-3)
Supplement: Supplementary file 3 — Supplementary Material 3 [file 12912_2023_1255_MOESM3_ESM.docx]

**Additional file 3** (.docx) - Other statements

The results obtained in some statements about the contents of training programmes are presented. Statements focus on mental health problems and mental health first aid actions.

**Contents of Training Programmes:** Mental Health Problems

| **Statements** | **Responses** | **n (%)** | |
| --- | --- | --- | --- |
|  |  | **Round 1** | **Round 2** |
| Cognitive problems to be addressed in training programmes include memory problems (impaired memory). | Agreement | 48 (62%) | 52 (85%)* |
|  | Neutral | 20 (26%) | 6 (10%) |
|  | Disagreement | 10 (13%) | 3 (5%) |
| Cognitive problems to be addressed in training programmes include attention problems (impaired attention, impaired concentration). | Agreement | 66 (85%)* |  |
|  | Neutral | 9 (12%) |  |
|  | Disagreement | 3 (4%) |  |
| Cognitive problems to be addressed in training programmes include language problems (dyslexia, dysphasia). | Agreement | 46 (59%) | 47 (77%)* |
|  | Neutral | 22 (28%) | 9 (15%) |
|  | Disagreement | 10 (13%) | 5 (8%) |
| Cognitive problems to be addressed in training programmes include problems of perception (hallucination). | Agreement | 52 (67%) | 53 (87%)* |
|  | Neutral | 12 (15%) | 5 (8%) |
|  | Disagreement | 14 (18%) | 3 (5%) |
| Cognitive problems to be addressed in training programmes include thinking problems (impaired thinking, impaired learning, suicidal ideation, obsession, delirium, dementia, confusion). | Agreement | 65 (83%)* |  |
|  | Neutral | 10 (13%) |  |
|  | Disagreement | 3 (4%) |  |
| Cognitive problems to be addressed in training programmes include impaired beliefs (negative self-image, disturbed personal identity, disturbed body image, low self-esteem, excessive self-esteem). | Agreement | 74 (95%)* |  |
|  | Neutral | 2 (3%) |  |
|  | Disagreement | 2 (3%) |  |
| Behavioural problems to be addressed in training programmes include self-care problems (self-care deficit). | Agreement | 75 (96%)* |  |
|  | Neutral | 2 (3%) |  |
|  | Disagreement | 1 (1%) |  |
| Behavioural problems to be addressed in training programmes include problems with compulsions (compulsive behaviour). | Agreement | 69 (88%)* |  |
|  | Neutral | 7 (9%) |  |
|  | Disagreement | 2 (3%) |  |
| Behavioural problems to be addressed in training programmes include eating problems (compulsive eating behaviour, impaired eating behaviour [anorexia], bulimia). | Agreement | 74 (95%)* |  |
|  | Neutral | 1 (1%) |  |
|  | Disagreement | 3 (4%) |  |
| Behavioural problems to be addressed in training programmes include problems in the pattern of exercise (impaired exercise behaviour, excessive physical exercise). | Agreement | 64 (82%)* |  |
|  | Neutral | 9 (12%) |  |
|  | Disagreement | 5 (6%) |  |
| Behavioural problems to be addressed in training programmes include substance use problems (alcohol abuse, drug abuse, tobacco abuse). | Agreement | 73 (94%)* |  |
|  | Neutral | 3 (4%) |  |
|  | Disagreement | 2 (3%) |  |
| Behavioural problems to be addressed in training programmes include problems with gambling, video games and internet ([gambling] abuse, [video games] abuse, [internet] abuse). | Agreement | 74 (95%)* |  |
|  | Neutral | 2 (3%) |  |
|  | Disagreement | 2 (3%) |  |
| Behavioural problems to be addressed in training programmes include aggressiveness problems (aggressive behaviour, aggressive behaviour [harassment], violence, [cybernetic] violence, [sexual] violence). | Agreement | 76 (97%)* |  |
|  | Neutral | 1 (1%) |  |
|  | Disagreement | 1 (1%) |  |
| Behavioural problems to be addressed in training programmes include self-injurious problems (self-destructive behaviour, self-mutilation, attempted suicide). | Agreement | 73 (94%)* |  |
|  | Neutral | 3 (4%) |  |
|  | Disagreement | 2 (3%) |  |
| Behavioural problems to be addressed in training programmes include problems in sexual behaviour (impaired sexual behaviour [paraphilias]). | Agreement | 58 (74%) | 40 (66%) |
|  | Neutral | 10 (13%) | 12 (20%) |
|  | Disagreement | 10 (13%) | 9 (15%) |
| Behavioural problems to be addressed in training programmes include sleep problems (impaired sleep, insomnia). | Agreement | 72 (92%)* |  |
|  | Neutral | 4 (5%) |  |
|  | Disagreement | 2 (3%) |  |
| Behavioural problems to be addressed in training programmes include problems in the organisation of behaviours (disorganised behaviour). | Agreement | 65 (83%)* |  |
|  | Neutral | 9 (12%) |  |
|  | Disagreement | 4 (5%) |  |
| Behavioural problems to be addressed in training programmes include problems with the frequency of behaviours (hyperactivity). | Agreement | 63 (81%)* |  |
|  | Neutral | 11 (14%) |  |
|  | Disagreement | 4 (5%) |  |
| Emotional problems to be addressed in training programmes include the existence of negative emotions (anxiety, nervousness, sadness, depressed mood, grief, loneliness, ambivalence, euphoria, fear, trauma, stress, fatigue, exhaustion, distress, despair, jealousy, guilt, frustration, insecurity, envy, anger, powerlessness, suffering, shame). | Agreement | 75 (96%)* |  |
|  | Neutral | 2 (3%) |  |
|  | Disagreement | 1 (1%) |  |
| Emotional problems to be addressed in training programmes include lack of positive emotions (lack of hope, lack of trust, lack of pride, lack of pleasure). | Agreement | 75 (96%)* |  |
|  | Neutral | 2 (3%) |  |
|  | Disagreement | 1 (1%) |  |
| Relational problems to be addressed in training programmes include problems of socialisation (social isolation, impaired socialisation). | Agreement | 76 (97%)* |  |
|  | Neutral | 0 (0%) |  |
|  | Disagreement | 2 (3%) |  |
| Relational problems to be addressed in training programmes include communication problems (impaired communicating act). | Agreement | 71 (91%)* |  |
|  | Neutral | 3 (4%) |  |
|  | Disagreement | 4 (5%) |  |

Legend: ***** - Consensus obtained.

**Contents of Training Programmes:** Mental Health First Aid Actions

| **Statements** | **Responses** | **n (%)** |
| --- | --- | --- |
|  |  | **Round 1** |
| In the step “Approach the person and assess the situation”, an aider can: introduce yourself; explain the purpose of the aider's presence; ensure confidentiality; express availability (to listen, be present and help); be present; actively listening with an expression of interest, respect, understanding and without judgement; assess the person's cultural background; observe behaviour; coordinate behaviour with the person being helped; physically approach the person, ensuring their safety; accompany the person to a quiet, safe, comfortable place that allows privacy; ask about the situation; ask about the problem, causes, aggravating and mitigating factors and consequences. | Agreement | 70 (90%)* |
|  | Neutral | 6 (8%) |
|  | Disagreement | 2 (3%) |
| In the step “Assist and encourage the person to use self-help strategies”, an aider can: assist in identifying the mental health problem; inform about the mental health problem; assist in identifying adaptive strategies; encourage the use of adaptive strategies; praise adaptive strategies already in use; encourage abandonment of maladaptive strategies. | Agreement | 74 (95%)* |
|  | Neutral | 1 (1%) |
|  | Disagreement | 3 (4%) |
| In the step “Assist and encourage the person to seek formal and informal help”, an aider can: assist in the seeking for informal and formal help; inform about the role of help resources; accompany in the seeking for informal and formal help; asking for help in an extreme situation; encourage the seeking for informal and formal help. | Agreement | 78 (100%)* |
|  | Neutral | 0 (0%) |
|  | Disagreement | 0 (0%) |
| In the step “Take care of oneself (first aider)”, an aider can use adaptive self-help strategies and, if necessary, can seek help for themselves. | Agreement | 76 (97%)* |
|  | Neutral | 2 (3%) |
|  | Disagreement | 0 (0%) |

Legend: ***** - Consensus obtained.
