# Supplementary material for: Nurses’ promotion of Mental Health First Aid Training Programmes for upper secondary students: a modified Delphi approach
Source: BMC Nurs. 2023 Mar 31;22:91. doi: 10.1186/s12912-023-01255-3 (PMC10064774; doi:10.1186/s12912-023-01255-3)
Supplement: Supplementary file 4 — Supplementary Material 4 [file 12912_2023_1255_MOESM4_ESM.docx]

**Additional file 4** (.docx) - Content analysis

Content analysis of justifications for agreement/disagreement with a statement is presented. The statement is “Behavioural problems to be addressed in training programmes include problems in sexual behaviour (impaired sexual behaviour [paraphilias])”.

**Content analysis of the justifications given**

**for agreeing with the statement without consensus**

| **Code** | **Category** | **Theme** |
| --- | --- | --- |
| Importance of sexuality in adolescence (E57) | Sexuality in adolescence | Promotion of competences to act on a problem relevant to the participants |
| Adolescents' interest (E68) |  |  |
| Relevance of the problem (E3, E5, E38) | Relevance of the problem |  |
| Current problem (E44) | Prevalence of the problem |  |
| Globalization of the problem (E44) |  |  |
| High prevalence of the problem (E12) |  |  |
| Existence of the problem (E73) |  |  |
| Attendance in the school context (E8) |  |  |
| Importance of dysfunctionality caused by the problem (E24) | Dysfunctionality caused by the problem |  |
| Consequences of the problem (E6) |  |  |
| Alert to the existence of the problem (E74) | Promotion of competencies to act on the problem |  |
| Promotion of knowledge about the problem (E55) |  |  |
| Promoting understanding of the problem (E10, E15, E49) |  |  |
| Promotion of recognition of the problem (E49, E73) |  |  |
| Promoting awareness of the problem (E30) |  |  |
| Promotion of intervention and referral of the problem (E30) |  |  |
| Promotion of intervention in the problem (E10) |  |  |
| Promotion of referral to professionals (E49) |  |  |

Note: Each expert's data was coded with the letter E and a number to preserve confidentiality.

**Content analysis of the justifications given**

**for disagreeing with the statement without consensus**

| **Code** | **Category** | **Theme** |
| --- | --- | --- |
| Dispensable in the type of programme (E28) | Specificity of the problem | Inappropriate learning for adolescents |
| Lack of relevance of the problem (E20) |  |  |
| Specificity of the problem (E40) |  |  |
| Low prevalence of the problem (E11) |  |  |
| Influence on the experience of adolescent sexuality (E66) | Sexuality in adolescence |  |
| Adolescents' lack of preparation to integrate information about the problem (E75) | Preparation to integrate information |  |

Note: Each expert's data was coded with the letter E and a number to preserve confidentiality.
